# Iron deficiency anemia and its association with cognitive function among adolescents in the Ashanti Region - Ghana

**DOI:** 10.1186/s12889-024-20640-4

**Published:** 2024-11-19

**Authors:** Francis Agyemang Yeboah, Joyce Bioh, Benjamin Amoani, Alfred Effah, Ebenezer Senu, Oscar Simon Olympio Mensah, Alex Agyei, Samuel Kwarteng, Samuel Kwame Sopuruchi Agomuo, Stephen Opoku, Samuel Kekeli Agordzo, Ebenezer Krampah Aidoo, Samuel Asamoah Sakyi

**Affiliations:** 1 Department of Molecular Medicine, School of Medicine and Dentistry, Kwame Nkrumah University of Science and Technology, Kumasi, Ghana; 2 Department of Chemical Pathology, Komfo Anokye Teaching Hospital, Kumasi, Ghana; 3 Department of Biomedical Science, School of Allied Health Sciences, University of Cape Coast, Cape Coast, Ghana; 4 Division of Clinical Immunology and Rheumatology, University of Alabama at Birmingham, Birmingham, USA; 5 Department of Medical Laboratory Technology, Accra Technical University, Accra, Ghana; 6 Department of Biological Sciences, School of Natural Sciences and Mathematics, The University of Texas at Dallas, Richardson, Texas, USA

**Keywords:** Iron deficiency, anemia, Cognitive function, Adolescents

## Abstract

**Background:**

Iron deficiency anemia (IDA) remains a global health concern, and has been associated with cognitive decline. However, very few studies have explored the association between IDA and cognitive function among Ghanaians. We assessed the association between IDA and cognitive function among adolescents in the Ashanti region, Ghana.

**Methods:**

This cross-sectional study involved 250 adolescents from Kumasi, Ghana. Sociodemographic and dietary data were obtained using a well-structured questionnaire. Blood samples were drawn for estimation of ferritin and complete blood count. The Test of Non-verbal Intelligence (TONI-4) was used to assess cognitive function. Binary logistic regression was used to determine the predictors of cognitive function.

**Results:**

The prevalence of IDA was 30.4%, which was higher among adolescents with poor cognitive performance test scores (CPTS) (71%). Being female [aOR = 0.32, 95% CI (0.10–0.99), *p* = 0.0480)], father having junior high education [aOR = 0.08, 95% CI (0.02–0.45), *p* = 0.0040)], being in a category B school [aOR = 0.26, 95% CI (0.09–0.81), *p* = 0.0200)] and C [aOR = 0.08, 95% CI (0.02–0.40), *p* = 0.0020)] and non-fruit consumption [aOR = 0.18, 95% CI (0.06–0.52), *p* = 0.0010)], were significantly associated with lower likelihood of having very good cognitive function. Moreover, ferritin (*r* = 0.451, *p* < 0.001) and hemoglobin (*r* = 0.402, *p* < 0.001) demonstrated a moderate positive correlation with CPTS.

**Conclusion:**

The prevalence of IDA is high in our study population and was linked with poor cognitive function. Adolescents with IDA had low cognitive performance test scores. High levels of hemoglobin and ferritin showed a moderate correlation with higher cognitive performance. These findings suggest that adolescents’ cognitive function may be moderately influenced by IDA, highlighting the potential impact of iron status on cognitive outcomes.

**Supplementary Information:**

The online version contains supplementary material available at 10.1186/s12889-024-20640-4.

## Introduction

The World Health Organization (WHO) defines human adolescence as age 10–19 years [1], which is a crucial time for transition from childhood to adulthood. This period is marked by rapid development, early learning, and mental developmental changes that can affect future behavior and health status of individuals [2, 3]. This rapid growth and development calls for a higher need of micro and macronutrients during the period [2].

Due to the rapid pubertal growth that results in a dramatic rise in muscle mass, quantity of blood, and red cell mass, iron requirements for myoglobin in muscles, hemoglobin in the blood and the overall iron needs of the body during this significant period peaks [4]. Total iron intake increases two to three folds from that of children’s daily intake of 0.7–0.9 mg to as high as 1.37–1.88 mg, and 1.40–3.27 mg daily in both adolescent boys and girls respectively [4]. In light of this, adolescents require twice as much iron than children do. Other established factors like limited iron diets, monthly blood loss in adolescent females, high incidence of diseases and parasitic infections, early marriages per societal beliefs and teenage pregnancies place adolescents at high risk for developing iron deficiencies, particularly in females [5].

Iron deficiency anemia (IDA) is the most common form of anemia worldwide and is estimated to be the cause of up to 50% of anemia cases [6]. Prevalence rate of IDA in adolescents is approximately 9–40% depending on the population studied [7]. Generally, adolescents in developing countries are more prone to iron deficiencies due to low socio-economic status [8]. Almost one-quarter of adolescents are affected with anemia in developing countries [9]. In Ghana anemia continues to be highly prevalent (45.1%) [10]. A recent study by Ampiah et al., also reported significant incidence of anemia among adults, pregnant women, and teenagers [11]. Anemia is characterized by low levels of hemoglobin, < 12 g/dl which causes impaired motor and cognitive development in children [12] with decreased work productivity in adulthood due to reduced work capacity.

Earlier research on cognition focused solely on young children, but cognitive development is now known as a lifetime process concurring by both genetic and environmental conditions [13]. It is proven that IDA, especially when severe, may predispose adolescents to complications like cognitive dysfunction [14]. IDA impairs the functions of iron-dependent enzymes such as tyrosine and tryptophan hydroxylases reducing the levels of neurotransmitters (serotonin, noradrenaline and dopamine) in the brain thereby affecting neurocognitive development [15]. Iron is involved in many fundamental biological processes in the brain including oxygen transportation, DNA synthesis, mitochondrial respiration and myelin synthesis [16]. IDA can disrupt iron homoeostasis inducing cellular damage through hydroxyl radical production, which can cause the oxidation and modification of lipids, proteins, carbohydrates and DNA [17] manifesting initially as cognitive impairment. Additionally, it is well recognized that even with iron therapy, such neurological deficit is not fully recoverable [18].

The majority of studies on IDA, in particular, concentrated on the early years of life. Current studies show a declining trend of IDA in kids and young children due to better food intake and awareness, IDA in teenagers continues to rise due to underlying factors as previously stated [19]. Although previous studies have reported high prevalence of IDA among Ghanaian adolescents, only few have assessed the association between IDA and cognitive function. This study evaluated iron deficiency anemia and its association with cognitive function among adolescents of selected Senior High Schools in the Ashanti Region, Kumasi-Ghana.

## Materials and methods

### Study design

This cross-sectional study was conducted among healthy adolescents from selected public senior high schools within the Ashanti Region. The research collaborated with the Transfusion Medicine Unit at the Komfo Anokye Teaching Hospital (KATH), Kumasi-Ghana to recruit the study participants, between September and October, 2022 through blood donation campaigns run by the transfusion unit.

### Sample size estimation and study population

The Cochran formula with a known previous study prevalence rate of 17.1% [20], was used to obtain a minimum sample size of 217. However, a total of 250 participants were recruited to improve statistical power. The 250 participants comprised voluntary adolescent boys and girls (blood donors) between the ages of 16 and 19yrs with no history of any chronic illness including sickle cell disease or infection.

### Inclusion and exclusion criteria

Both male and female school going adolescents with no history of any chronic diseases or infection were enrolled. However, adolescents who refused to consent to the study, or had history of any known chronic illness and also on any form of iron therapy as well as the females in their menstrual period were excluded from the study.

### Data collection

The data were collected using a structured, pretested, self-administered questionnaire adapted from a previous study and modified to suit the objectives of this study [21]. Participants completed structured questionnaires covering areas of their socio-demographic data, medical history, dietary habits/patterns, as well as their learning and sleeping patterns. In addition, menstrual characteristics were self-reported specifically among female adolescents to assess their menstrual history.

### Anthropometric measurements

Anthropometric measurements for this study included weight and height. Participants’ weights were recorded to the nearest 0.1 kg (kg) using a body weight scale (Seca GmbH & Co. KG., Germany), with belts and shoes removed. Height was measured to the nearest 0.1 centimeter (cm) using a wall-mounted measuring tape (5 M/16FT measuring tape) in cm, with participants standing upright, feet together, and hands at their sides.

### Blood sample collection and laboratory assays

Venous blood (5 mL) was collected from each participant under complete aseptic conditions into a serum separator tube for biochemical indices and an EDTA-coated tube for hematological indices. All samples taken were analyzed at the Clinical Biochemistry and Haematology Department of KATH, Kumasi-Ghana. At the lab, serum separator samples were centrifuged using a thermos scientific Heraeus Megafuge 8 at 4000 rpm for 4 min. Serum/ supernatant was separated into a cryo-tube and stored at -80 °C freezer. Complete blood count (CBC) test was performed using the EDTA samples with Sysmex XN 2000. Biochemical assay for serum ferritin estimation was performed using Biosystems A25 (Biosystems, Spain) based on manufacturer’s instructions.

### Nonverbal intelligence test (TONI- 4)

Test of Nonverbal Intelligence: fourth edition (TONI-4) which has been used in other studies and proven to have good reliability and validity [21], was used to assess cognitive function. The test measures intelligence, aptitude, abstract reasoning, visual-spatial processing and problem-solving ability. The test was completely nonverbal or language-free, ideal for evaluating those with questionable or limited language ability. Each form of the TONI-4 contains 50 items arranged from easy to difficult order. Due to time constraints and the concurrent blood donation exercise, two items were selected from every tenth question, as these items were representative of the test’s overall difficulty progression. Thus, study participants were given a set of printed shapes and patterns to determine the relationship between them within 10 min period. The aim was to ascertain students’ critical thought and cognitive flexibility to determine the accuracy and quickness with which they can detect and analyze the connections between a set of shapes and patterns. There were 10 questions in all and each scored 10 marks making 100% total score. With reference to the TONI-4 test scores, ≥ 60% is considered very good cognitive performance, 40 – 59% as good and < 40% as poor cognitive performance. However, to predict “very good” using binary logistic regression model, “poor” and “good” cognition categories were combined into a single category (not very good), resulting in a binary outcome (not very good vs. very good).

### Validity and reliability of the abridged version of TONI-4

The abridged version was given to five psychologists to independently assess its validity. All five psychologists concluded that the 10 selected items adequately represent the nonverbal intelligence construct measured by the full version and deemed it appropriate to use the 50-item version’s cutoffs. Moreover, the abridged version demonstrated high internal consistency with a Cronbach’s alpha (ɑ) of 0.83.

### Statistical analysis

Data collected were analyzed using Statistical Package for Social Science (SPSS) windows version 26.0 and R programming language. Continuous variables were presented as mean ± standard deviation (SD) or median (interquartile range (IQR)) based on variable normality. Categorical variables were presented as frequency and percentages (%). The independent sample t test or Mann Whitney U test was used to compare continuous variables between various study groups depending on the normality. The Chi-square test was used to determine the association between cognitive function and IDA, whiles binary logistic regression was used to determine the independent predictors of IDA and cognitive function. Correlation was performed using Pearson and Spearman correlation coefficient (r). *P* < 0.05 was considered statistically significant.

## Results

### Sociodemographic characteristics of school-going adolescents enrolled in the study

Of the 250 participants enrolled in this study, the mean age was 17.5 ± 0.7 years, with the majority being female (57.2%). Most (72%) of the participants were residing in the urban areas with approximately 79% being Christians. Considering parents education, most of the parents had basic education (primary and junior high). About 56% of the participants were from a family of 3 to 5 children, followed by those from families of 6 or more children (36%), whilst few were from families of 1 to 2 children (8.4%). Moreover, approximately three quarter (78%) of the participants had good (> 6 h) sleeping hours whilst few (22%) had poor sleeping hours. Also, most (76%) of the school-going adolescents recruited in this study had normal body mass index (BMI), followed by overweight (16%), underweight (6.4%) and obese (1.6%). Additionally, more (39.2%) of the participants were from category B schools, followed by C (22.8%), A (24%) whilst few (14%) were from category D schools (Table 1).

**Table 1:** Sociodemographic characteristics of study participants

Variable	Frequency (*n* = 250)	Percentage (%)
**Age Group (years)**	17.5 ± 0.7	
**Gender**		
Male	107	42.8
Female	143	57.2
**Residence**		
Rural	70	28.0
Urban	180	72.0
**Religion**		
Christian	198	79.2
Muslim	49	19.6
Traditionalist	3	1.2
**Father’s Education**		
Primary	44	17.6
Junior High	83	33.2
Secondary	86	34.4
Tertiary	37	14.8
**Mother’s Education**		
Primary	59	23.6
Junior High	109	43.6
Secondary	61	24.4
Tertiary	21	8.4
**Family Size**		
Small (1–2)	21	8.4
Medium (3–5)	139	55.6
Large (≥ 6)	90	36.0
**Sleeping Duration (hours)**		
Poor (< 6)	55	22.0
Good (> 6)	195	78.0
**BMI Category**		
Underweight	16	6.4
Normal	190	76.0
Overweight	40	16.0
Obese	4	1.6
**School Category**		
Grade A	60	24.0
Grade B	98	39.2
Grade C	57	22.8
Grade D	35	14.0

Data represented as frequency and percentages, BMI: Body Mass Index

### Anthropometric measurements, hematological characteristics of study participants stratified by gender

Weight did not differ between gender (*p* = 0.3110). However, there was a significant difference in height (*p* < 0.0001) and BMI (*p* < 0.0001) between male and female participants. Similarly, all the hematological and biochemical parameters such as red blood cell (RBC) (*p* < 0.0001), hemoglobin (HGB) (*p* < 0.0001), hematocrit (HCT) (*p* < 0.0001), mean corpuscular volume (MCV) (*p* < 0.0001), mean corpuscular hemoglobin (MCH) (*p* < 0.0001), mean Corpuscular hemoglobin concentration (MCHC) (*p* < 0.0001) and ferritin (*p* < 0.0001), were significantly different between male and female participants. Moreover, the cognitive performance test score (CPTS) in this study was 50 (40–60) and 40 (30–50) for males and females respectively, which was statistically different (*p* < 0.0001) (Table 2).

**Table 2:** Anthropometric, biochemical, hematological characteristics, and Cognitive Performance Test scores (CPTS) of adolescents stratified by gender

Variable	Total (*n* = 250)	Male (*n* = 107)	Female (*n* = 143)	*p*-value
**Anthropometric**				
Weight (Kg)	59.00 (55.00–65.00)	60.00 (55.00–65.00)	58.00 (54.00–66.00)	0.3110
Height (m)	1.65 (1.58–1.71)	1.72 (1.65–1.75)	1.60 (1.55–1.65)	**< 0.0001**
Body Mass Index (BMI)	21.97 (20.20–24.23)	20.57 (19.03–22.27)	22.97 (21.63–25.51)	**< 0.0001**
**Hematology**				
Ferritin (ng/ml)	30.75 (14.05–56.97)	51.23 (30.51–101.78)	18.06 (10.98–37.30)	**< 0.0001**
RBC* (10^6^/µl)	4.66 ± 0.53	4.98 ± 0.48	4.42 ± 0.43	**< 0.0001**
HGB (g/dL)	12.35 (11.40–14.23)	14.50 (13.40–15.20)	11.60 (10.80–12.20)	**< 0.0001**
HCT (%)	36.85 (33.90–41.40)	41.80 (39.00–44.10)	34.50 (32.5–36.70)	**< 0.0001**
MCV (fL)	81.35 (77.23–86.18)	83.30 (80.50–87.80)	79.80 (72.80–84.20)	**< 0.0001**
MCH (pg)	28.10 (26.18–30.10)	29.20 (27.60–30.50)	27.30 (24.50–29.10)	**< 0.0001**
MCHC (g/dL)	34.10 (33.18–35.10)	34.50 (34.00–35.60)	11.60 (32.4–34.70)	**< 0.0001**
**CPTS**	40.00 (30.00–55.00)	50.00 (40.00–60.00)	40.00 (30.00–50.00)	**< 0.0001**

Data presented as median (IQR), * mean ± SD, p-values obtained by Mann Whitney-U test and independent Sample T test. CPTS = cognitive performance test score, MCV: Mean Corpuscular Volume, MCH: Mean Corpuscular Hemoglobin, MCHC: Mean Corpuscular Hemoglobin Concentration, RBC: Red Blood Cell, HCT: Hematocrit, HGB: Hemoglobin. p-value < 0.05 was considered statistically significant

### Prevalence of Iron Deficiency Anemia (IDA) among school-going adolescents

**Fig. 1:**
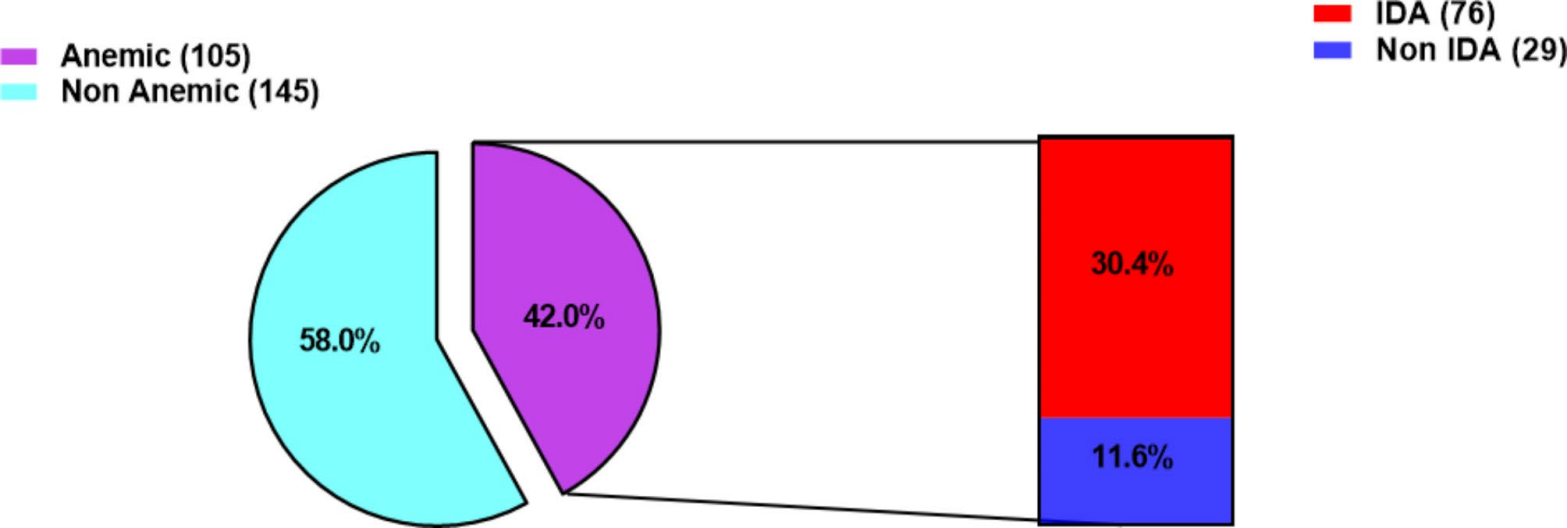
Prevalence of anemia and iron deficiency anemia among adolescents recruited in the study

Among the 250 participants, the proportion of anemia and iron deficiency (ID) were 42% and 47.2% respectively. Moreover, the overall prevalence of iron deficiency anemia in this study was 30.4%, with 11.6% anemia without iron deficiency (Fig. 1).

### Cognitive performance test scores of participants

**Fig. 2:**
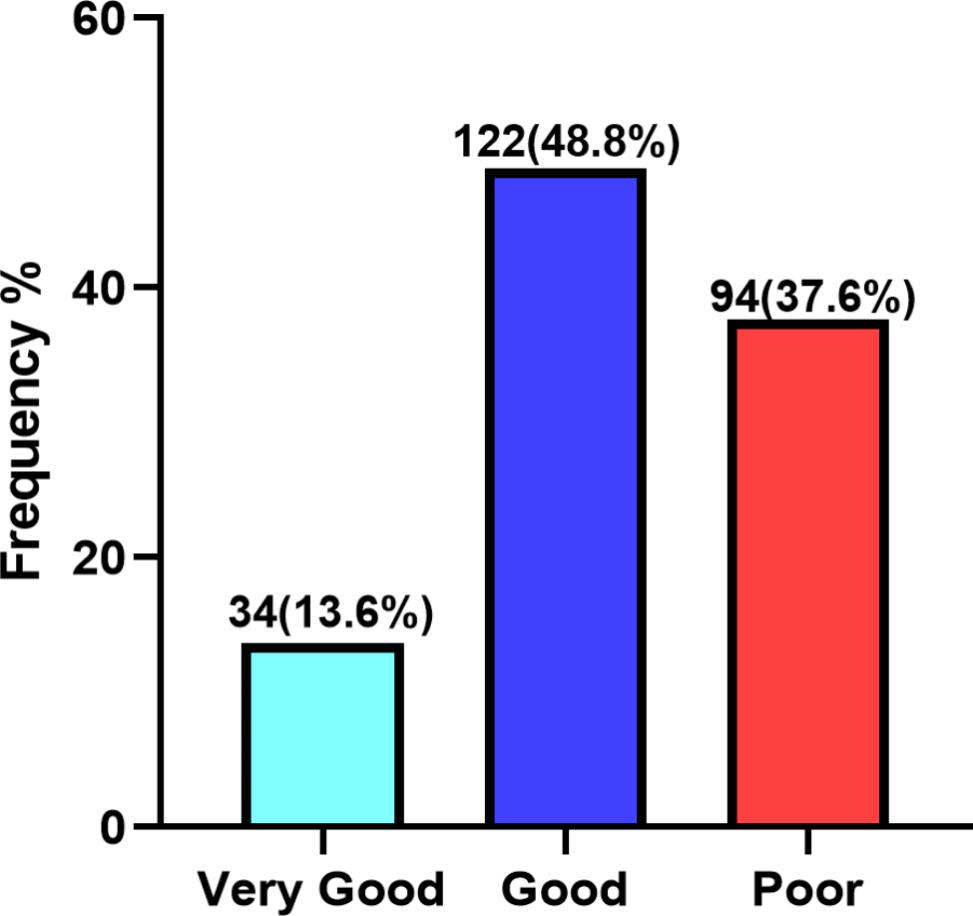
Cognitive test scores of study participants, very good (≥ 60%), good (40–59%) and poor (< 40%)

The mean scores of cognitive performance test were 75.9 (± 6.6), 48.2 (± 7.4) and 24.2 (± 7.5) for very good, good and poor respectively. Among the 250 study participants, majority (48.8%) had good, followed by poor (37.6%), whilst few (13.6%) had very good cognitive function (Fig. 2).

### Sociodemographic predictors of iron deficiency anemia among adolescents

After adjusting for possible confounders such as age and gender in a multivariable logistic regression model, being female [aOR = 31.71, 95% CI (8.46–118.92), *p* < 0.000), mother having primary [aOR = 8.14, 95% CI (1.15–57.59), *p* = 0.0360)] or secondary education [aOR = 6.09, 95% CI (1.04–35.64), *p* = 0.0450)] and being obese [aOR = 11.10, 95% CI (2.26–54.60), *p* = 0.0030)] were independently associated with higher odds of getting IDA. On the other hand, having a father with junior high [aOR = 0.23, 95% CI (0.06–0.83), *p* = 0.0240] or secondary education [aOR = 0.24, 95% CI (0.07–0.78), *p* = 0.0180)] as the highest educational level was independently associated with lower chances of IDA among the study participants (Table 3).

**Table 3:** Sociodemographic predictors of IDA among study participants

Variable	IDA (*n* = 76)	cOR (95% CI)	*p*-value	aOR (95% CI)	*p*-value
**Gender**					
Male (Ref)	5 (6.6)	1.00	-	1.00	-
Female	71 (93.4)	20.12 (7.74–52.32)	**< 0.0001**	31.71 (8.46–118.92)	**< 0.0001**
**Residence**					
Rural	21 (27.6)	0.97 (0.53–1.78)	0.9320	1.74 (0.77–3.94)	0.1810
Urban (Ref)	55 (72.4)	1.00	-	1.00	-
**Religion**					
Christian	57 (75.0)	0.20 (0.02–2.27)	0.9500	0.17 (0.01–2.96)	0.2230
Muslim	17 (22.4)	0.27 (0.02–3.15)	0.2930	0.38 (0.02–7.64)	0.5270
Traditionalist (Ref)	2 (2.6)	1.00	-	1.00	-
**Father’s Education**					
Primary	18 (23.7)	1.14 (0.46–2.79)	0.7780	0.34 (0.08–1.46)	0.1450
Junior High	23 (30.3)	0.63 (0.28–1.43)	0.2690	0.23 (0.06–0.83)	**0.0240**
Secondary	21 (27.6)	0.53 (0.23–1.21)	0.1330	0.24 (0.07–0.78)	**0.0180**
Tertiary (Ref)	14 (18.4)	1.00	-	1.00	-
**Mother’s Education**					
Primary	23 (30.3)	3.83 (1.01–14.49)	**0.0480**	8.14 (1.15–57.59)	**0.0360**
Junior High	32 (42.1)	2.49 (0.69–9.06)	0.1650	4.39 (0.75–25.72)	0.1010
Secondary	18 (23.7)	2.51 (0.66–9.60)	0.1780	6.09 (1.04–35.64)	**0.0450**
Tertiary (Ref)	3 (3.9)	1.00	-	1.00	-
**Family Size**					
Small (1–2) (Ref)	6 (7.9)	1.00	-	1.00	-
Medium (3–5)	43 (56.6)	1.12 (0.41–3.08)	0.8270	1.29 (0.35–4.72)	0.7010
Large (≥ 6)	27 (35.5)	1.07 (0.38–3.06)	0.8970	0.98 (0.25–3.89)	0.9740
**Sleeping Duration (hours)**					
Poor (< 6)	19 (25.0)	1.28 (0.68–2.41)	0.4500	1.42 (0.64–3.17)	0.3930
Good (> 6) (Ref)	57 (75.0)	1.00	-	1.00	-
**BMI Category**					
Underweight (Ref)	2 (2.6)	1.00	-	1.00	-
Normal	54 (71.1)	2.78 (0.61–12.64)	0.1860	3.78 (0.98–14.50)	0.0530
Overweight	18 (23.7)	5.73 (1.15–28.57)	**0.0330**	2.88 (0.71–11.67)	0.1390
Obese	2 (2.6)	7.00 (0.60–81.68)	0.1210	11.10 (2.26–54.60)	**0.0030**
**School Category**					
Grade A	5 (6.6)	1.00	-	1.00	-
Grade B	36 (47.4)	6.39 (2.34–17.42)	**< 0.0001**	0.19 (0.02–1.89)	0.1570
Grade C	18 (23.7)	5.08 (1.74–14.84)	**0.0030**	0.18 (0.02–2.15)	0.1770
Grade D	17 (22.4)	10.39 (3.36–32.17)	**< 0.0001**	0.06 (0.00–1.69)	0.0970

cOR = crude odd ratio, aOR = adjusted odd ratio, CI = confidence interval, p-value of < 0.05 was considered statistically significant

### Correlation between anthropometric measures, hemoglobin and ferritin

**Fig. 3:**
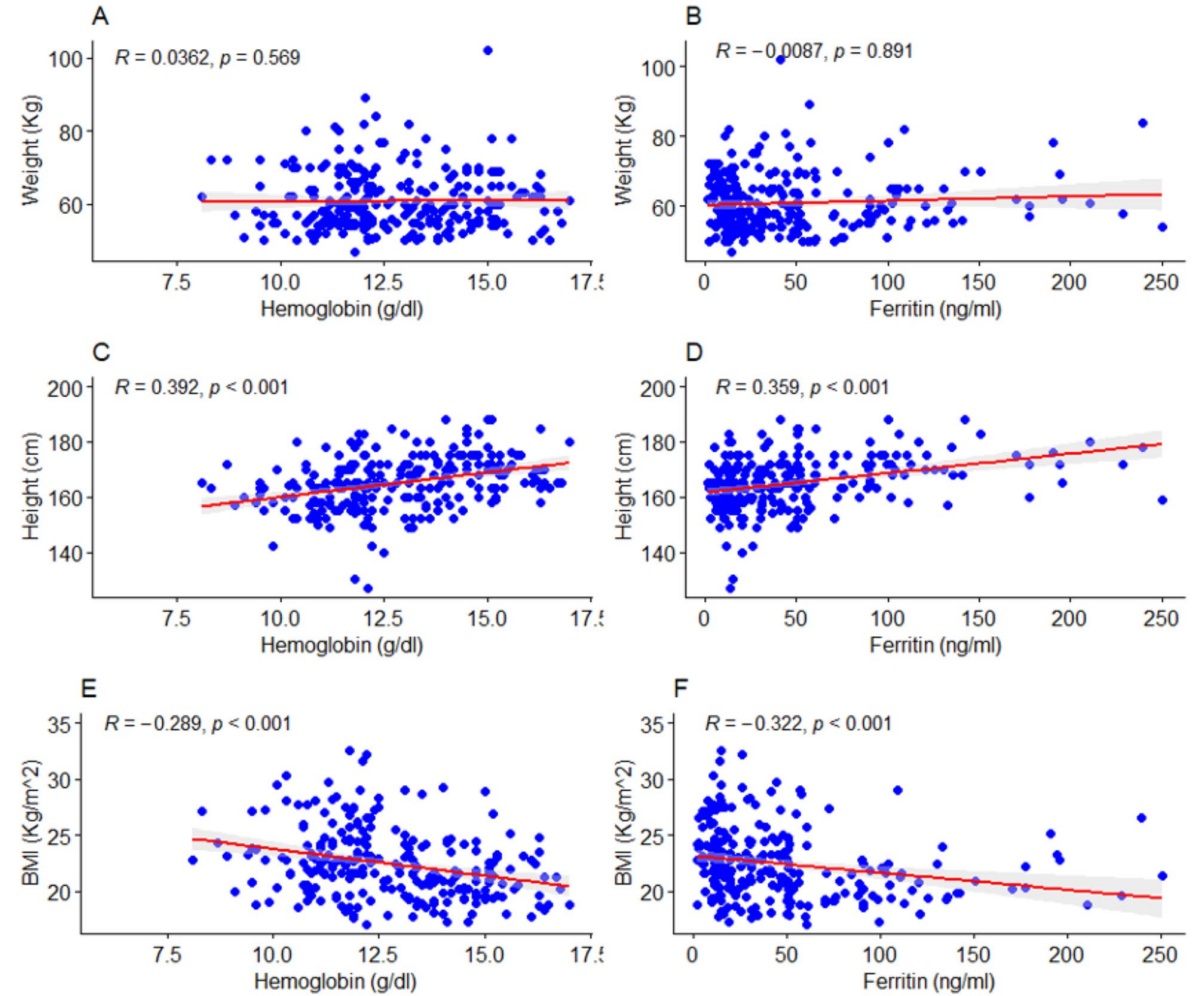
Spearman correlation between weight (**A-B**), height (**C-D**), BMI (**E-F**) and levels of hemoglobin and ferritin

The study revealed significant positive correlation between height and hemoglobin (*r* = 0.392, *p* < 0.001) (Fig. 3C). Similarly, a significant positive correlation was also observed between height and ferritin (*r* = 0.359, *p* < 0.001) (Fig. 3D). BMI was negatively correlated with hemoglobin (*r* = − 0.289. *p* < 0.001), and ferritin (*r* = − 0.322, *p* < 0.001) (Fig. 3E, F). However, **t**here was no significant correlation between weight and hemoglobin (*r* = 0.0362, *p* = 0.569) as well as ferritin levels (*r* = − 0.0087, *p* = 0.891) (Fig. 3A, B).

### Predictors of cognitive performance

Following the adjustment of putative confounders in a multivariable logistic regression model, being female [aOR = 0.32, 95% CI (0.10–0.99), *p* = 0.0480)], father having junior high education [aOR = 0.08, 95% CI (0.02–0.45), *p* = 0.0040)], being in category B [aOR = 0.26, 95% CI (0.09–0.81), *p* = 0.0200)] and C [aOR = 0.08, 95% CI (0.02–0.40), *p* = 0.0020)] and non-fruit consumption [aOR = 0.18, 95% CI (0.06–0.52), *p* = 0.0010)], were the independent predictors of very good cognitive function (Table 4).

**Table 4:** Predictors of very good cognitive performance

Variable	Very Good (*n* = 34)	cOR (95% CI)	*p*-value	aOR (95% CI)	*p*-value
**Gender**					
Male (Ref)	24 (70.6)	1.00	-	1.00	-
Female	10 (29.4)	0.26 (0.12–0.57)	**0.0010**	0.32 (0.10–0.99)	**0.0480**
**Residence**					
Rural	7 (20.6)	0.63 (0.26–1.52)	0.3040	0.54 (0.19–1.56)	0.2560
Urban (Ref)	27 (79.4)	1.00	-	1.00	-
**Father’s Education**					
Primary	6 (17.6)	0.37 (0.12–1.14)	0.0830	0.67 (0.11–3.94)	0.6550
Junior High	3 (8.8)	0.09 (0.02–0.34)	**< 0.0001**	0.08 (0.02–0.45)	**0.0040**
Secondary	14 (41.2)	0.46 (0.19–1.14)	0.0930	0.48 (0.15–1.52)	0.2110
Tertiary (Ref)	11 (32.4)	1.00	-	1.00	-
**Mother’s Education**					
Primary	7 (20.6)	0.43 (0.12–1.55)	0.1960	3.62 (0.47–27.91)	0.2180
Junior High	14 (41.2)	0.47 (0.15–1.49)	0.2000	4.72 (0.94–23.67)	0.0590
Secondary	8 (23.5)	0.48 (0.14–1.69)	0.2540	1.62 (0.34–7.74)	0.5490
Tertiary (Ref)	5 (14.7)	1.00	-	1.00	-
**Family Size**					
Small (1–2) (Ref)	4 (11.8)	1.00	-	1.00	-
Medium (3–5)	19 (55.9)	1.69 (0.48–5.95)	0.4140	0.61 (0.15–2.57)	0.4990
Large (≥ 6)	11 (32.4)	1.14 (0.51–2.52)	0.7510	0.44 (0.09–2.25)	0.3230
**Sleeping Duration (hours)**					
Poor (< 6)	9 (26.5)	1.33 (0.58–3.05)	0.4990	1.51 (0.53–4.26)	0.4400
Good (> 6) (Ref)	25 (73.5)	1.00	-	1.00	-
**BMI Category**					
Underweight (Ref)	6 (17.6)	1.00	-	1.00	-
Normal	24 (70.6)	0.24 (0.08–0.72)	**0.0110**	0.91 (0.21–4.03)	0.9030
Overweight	4 (11.8)	0.19 (0.04–0.79)	**0.0220**	1.40 (0.19–10.63)	0.7440
Obese	0 (0.0)	0.00 (0.00–0.00)	0.9990	1.40 (0.00–0.00)	> 0.9999
**School Category**					
Grade A (Ref)	22 (64.7)	1.00	-	1.00	-
Grade B	10 (29.4)	0.20 (0.09–0.45)	**< 0.0001**	0.26 (0.09–0.81)	**0.0200**
Grade C	2 (5.9)	0.06 (0.01–0.28)	**< 0.0001**	0.08 (0.02–0.40)	**0.0020**
Grade D	0 (0.0)	0.00 (0.00–0.00)	0.9980	0.00 (0.00–0.00)	0.9980
**Fruits**					
Non-consumer	19 (55.9)	0.17 (0.08–0.38)	**< 0.0001**	0.18 (0.06–0.52)	**0.0010**
Low-consumer (Ref)	15 (44.1)	1.00	-	1.00	-
**Dairy Products**					
Non-consumer	5 (14.7)	0.03 (0.00–0.60)	**0.021**	0.14 (0.01–3.30)	0.2250
Low-consumer	28 (82.4)	0.45 (0.03–7.48)	0.579	1.43 (0.07–30.19)	0.8200
Consumer (Ref)	1 (2.9)	1.00	-	1.00	-
**Protein**					
Non-consumer	2 (5.9)	0.13 (0.01–1.72)	0.12	0.36 (0.02–6.59)	0.4890
Low-consumer	31 (91.2)	0.83 (0.09–7.70)	0.871	1.01 (0.09–11.98)	0.9940
Consumer (Ref)	1 (2.9)	1.00	-	1.00	-
**Difficulty in Learning**					
Yes	14 (41.2)	0.06 (0.02–0.13)	**< 0.0001**	0.56 (0.19–1.67)	0.3000
No (Ref)	20 (58.8)	1.00	-	1.00	-
**Retentive Memory**					
Yes (Ref)	32 (94.1)	1.00	-	1.00	-
No	2 (5.9)	0.02 (0.01–0.09)	**< 0.0001**	0.88 (0.09–8.86)	0.9120

cOR = crude odd ratio, aOR = adjusted odd ratio, CI = confidence interval, p-value of < 0.05 was considered statistically significant, bolded p-values are statistically significant

### Association between cognitive function and IDA

**Fig. 4:**
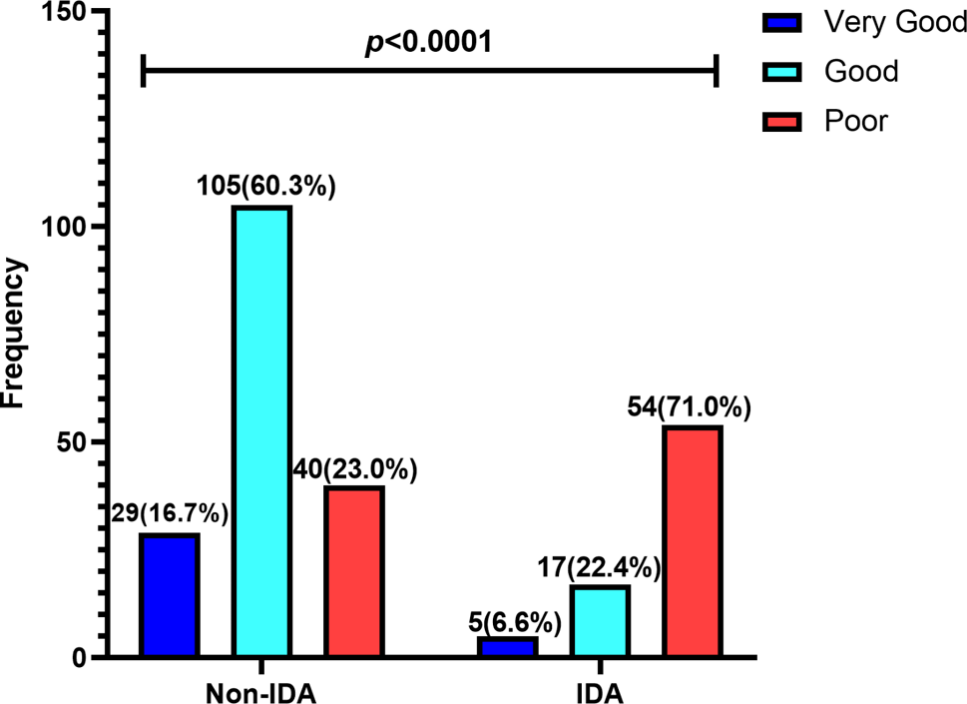
Cognitive performance test scores stratified by IDA status

In this study, IDA was recorded highest among adolescents with poor CPTS (71%) compared to those with good (22.4%) and very good (6.6%) CPTS. There was statistically significant association between cognitive function and IDA (*p* < 0.0001) (Fig. 4).

### Correlation between ferritin, hemoglobin and cognitive performance test score

**Fig. 5:**
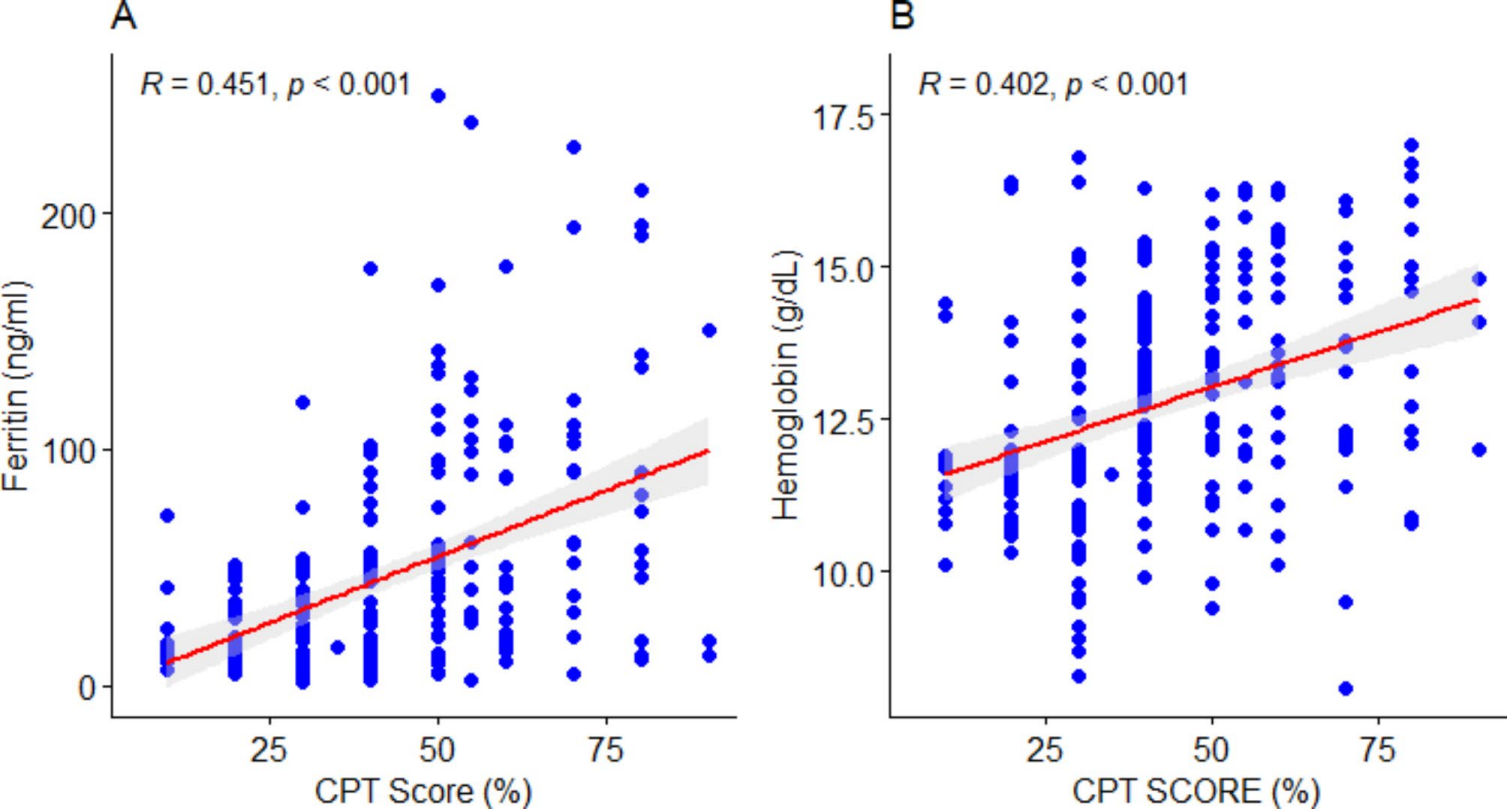
Correlation between (**A**) ferritin, (**B**) hemoglobin and cognitive performance test score (CPT)

The study found significant positive correlation between ferritin and CPTS (*r* = 0.451, *p* < 0.001) (Fig. 5A). Also, there was significant positive correlation between hemoglobin and CPTS (*r* = 0.402, *p* < 0.001) (Fig. 5B).

## Discussion

Despite the high prevalence of IDA among Ghanaian adolescents, only a few studies have assessed the association between IDA and cognitive function. This study evaluated iron deficiency anemia and its association with cognitive function among adolescents of selected Senior High Schools in the Ashanti Region, Kumasi-Ghana. We observed a high prevalence of IDA among our study participants, which was significantly higher in adolescents with poor CPTS. Female gender, father’s educational level, attendance at category B or C schools, and non-consumption of fruit were significantly associated with a lower likelihood of having very good cognitive function. Moreover, ferritin and hemoglobin demonstrated a moderate positive correlation with CPTS.

This current study revealed anemia, iron deficiency with or without anemia, and IDA prevalence as 42%, 47.2% and 30.4% respectively. This finding is consistent with a study conducted by Khan et al., (2023) who reported 51% and 34.3% for anemia and IDA among adolescents [22]. Undoubtedly, adolescents have persistently had high rates of IDA; therefore, campaigns to inform parents and guardians about the disease’s prevention and treatment are necessary. IDA prevalence among female participants (93.4%) was significantly higher as compared to their male colleagues (6.6%). This is in accordance with several studies which also reported a higher incidence of anemia approximately 50 – >90% in almost all age groups for females, than in males [23–25]. In developing and poor economies, females usually are given less food as to males with the assertion that males need more energy to grow [26]. Females also have the habit of skipping meals and taking in carbonated drinks and snacks prepared from refined cereals which are not good sources of iron [27]. This and other social discrimination towards the girl child, coupled with menstrual losses place the adolescent females at high risk of IDA than their male counterparts [26, 28]. Blood loss, more importantly gynecological blood loss, including heavy menses are found to be major risk factors that contribute to the development of IDA in adolescent females [29]. Studies conducted in different geographical regions may yield contrasting results due to variations in dietary habits, access to healthcare, and genetic factors as well as variations in the control for confounding factors such as diet, exercise, and other health conditions.

Consequently, high prevalence of IDA in adolescents is mainly related to low socio-economic status contributing to inadequate dietary intake of iron and poor bioavailability of iron consumed [30, 31]. Ghana is one of the nations with the highest burden of malnutrition leading to IDA, stunted growth, low serum retinol, and undernutrition [32]. For economic reasons, most adolescents are more concerned with filling their appetites than with the nutritional value of their food [33]. Additionally, in developing countries, low socio-economic conditions, lack of knowledge on good dietary practices and restricted access to food contribute to the high incidence of IDA [33]. Moreover, carbohydrate consumption was significantly related to IDA and this was the highest dietary intake among the studied population as compared to vegetables, dairy products, proteins and fruits. A study conducted in Ghana showed that animal foods per calorie have a higher influence on hemoglobin levels [34]. However, a typical Ghanaian meal is chiefly carbohydrate with little animal protein. IDA was highly recorded among non-vegetable consumers and non-dairy products consumers. This observation may be attributed to the lower iron intake and reduced iron absorption in non-vegetable and non-dairy consumers. Vegetables provide non-heme iron and key nutrients, such as vitamin C, which are essential for enhancing iron absorption [35, 36]. Diets lacking these food groups may therefore be insufficient in both iron content and the necessary cofactors required for optimal iron uptake, increasing the risk of IDA.

Furthermore, the study revealed that the majority of mothers of participants with IDA had primary or secondary education, both of which were significantly associated with a higher likelihood of IDA in their children. Consistently, many studies showed that adolescents who have parents with low educational levels, particularly mothers, are at an increased risk of developing anemia [31, 37]. Mothers with limited formal education are unlikely to have knowledge of iron-rich foods or even be able to read and comprehend a lot of food labels. In our setting, mothers are the main cooks in most homes, having much influence over the family’s food preparation, dietary habits, and intake. There was also a statistically significant association between IDA and school category. The study found that IDA was highly recorded among school category B, C and D than in A. According to the Ghana Education Service (GES) high school ratings, schools in category A are considered best as compared to B, C and D accordingly in the areas of building infrastructure, learning and all other social factors including their eating habits. Parents with high socioeconomic status can afford to enroll their children in category A schools within our society. This socioeconomic advantage enables students in category A schools to afford iron-rich foods compared to students in other school categories, which may explain the lower prevalence of IDA observed in category A schools in this study. School as an environmental factor plays an essential role on adolescents’ development and growth. Effective and high-quality schools support learner-centered guidance and learning environments which significantly enhance the cognitive, social, and emotional development of adolescents in such schools [38]. The study again found significant association between school category and Cognitive Performance Test scores. Participants at category A schools generally performed well as compared to B, C and D.

No significant relationship was found between the participants’ place of residence and IDA. This however contradicts other studies which reported associations between adolescents living in rural areas and anemia [2, 39]. Additionally, report from Sharief et al. revealed that about 62% of urban adolescent girls from the lower socio-economic group are estimated to be anemic [25]. This shows that IDA is associated with low socio-economic status independent of one’s residence which is in accordance with our study findings. Likewise, no significant association between one’s religion and IDA was found. Our finding is consistent with Agrawal and colleagues who found no significant relation of religion on IDA among adolescents [31]. It was observed that majority of the study participants came from larger families. Family size was not significantly related to IDA. In contrast, Ramzi et al. found a significant association between large family size and anemia, while Shaka and Wondimagegne indicated otherwise [2, 40]. According to data from a National Sleep Foundation survey of adolescents aged 17 to 18, 75% of them slept for less than 8 h per night [41] and the reasons for these sleeping disorders has also been found to be associated with IDA. IDA is known to cause sleep disturbance and restless leg syndrome [42]. However, our study found no statistically significant difference between sleeping habits of participants and IDA. This lack of association may be attributed to the potential biases in the questionnaires we used to evaluate sleep habits, which could have affected the accuracy of the data collected. Our findings however agree with Brahami et al., (2020) who studied the association of anemia and cognitive impairments among adolescent girls and found no association with insomnia, daytime sleepiness and sleep apnea score between anemic and non-anemic groups [43].

After adjusting for possible confounders such as age and gender in a multivariable logistic regression model, being female, mother having primary or secondary education and being obese were independently linked to an increased risk of developing IDA. Again, with the exception of weight, we observed statistically significant correlation between height, BMI, hematological indices, ferritin, CPTS and IDA. Iron is required for adequate erythropoietic function, oxidative metabolism and cellular immune responses and its deficiency decrease normal physiological functions. Our findings are expected and consistent with numerous other studies [25, 44, 45].

Regarding cognitive performance test scores, males scored significantly higher compared to females. This may be because the majority of adolescent girls in Ghana are confined to handling domestic duties, depriving them of opportunities to develop their cognitive skills outside of the classroom [46]. Menstrual blood loss could be suggestive of the lower cognitive performance, as it has been associated with IDA [47, 48]. Iron is essential for energy metabolism, oxygen transport and neurotransmitter production in the brain [49]. Therefore, regular loss of iron during menstruation may reduce iron reserves in females, which might affect their cognitive function. Moreover, a statistically significant association between father’s education, sleeping hours, school category and cognitive performance test score among the participants were found. Also, eating adequate vegetables, fruits, dairy products, proteins, fats and oils among participants significantly correlated positively with CPTS. Several micronutrients like B group vitamins and iron, as well as many polyphenols play a crucial role in cognitive health [50]. The lack of nutritional variety and restricted availability of animal-based foods on the menus of boarding schools could be a reason for low cognitive test score performance. Furthermore, no significant association was found between age, residence, family size, religion, and CPT scores among study participants. Age group of adolescents in this study was narrow (16–19 years) and hence could be the reason for no significant association with CPT scores. Good sleeping habits were associated with CPTS. This finding is consistent with a study in China where short sleep in young adults was associated with worse cognitive function [51]. Short sleep duration has been shown to impair several elements of neurocognitive functions [41].

We observed significant association between IDA and cognitive function. Our research indicates that non-anemic participants outperformed those with IDA on the CPT. Iron deficiency often arises from inadequate intake, chronic blood loss, or malabsorption. It negatively impacts cognition, behavior, and motor skills, making adequate iron essential for neurological development, particularly during adolescence [52, 53]. ID is known to affect the hippocampus region of the brain resulting in cognitive impairment, which might explain our observation [54].

Our study highlights the prevalence of IDA among adolescents and its associated impact on cognitive function. As one of the few studies contributing to the literature on the relationship between IDA and cognitive function among adolescents in Ghana, it provides valuable insights into this important public health issue. However, the study’s cross-sectional design presents limitations, as it prevents the evaluation of causal relationships between IDA and cognitive function. Despite this limitation, the findings emphasize the need for further research to explore these associations longitudinally.

## Conclusion

The prevalence of IDA is high in our study population and was linked with poor cognitive function. Adolescents with IDA had low cognitive performance test scores. High levels of hemoglobin and ferritin showed a moderate correlation with higher cognitive performance. These findings suggest that adolescents’ cognitive function may be moderately influenced by IDA, highlighting the potential impact of iron status on cognitive outcomes.

## Electronic supplementary material

Below is the link to the electronic supplementary material.

Supplementary Material 1

## Data Availability

All data generated or analyzed during this study can be requested from corresponding author.
