# Supplementary material for: Iron deficiency anemia and its association with cognitive function among adolescents in the Ashanti Region - Ghana
Source: BMC Public Health. 2024 Nov 19;24:3209. doi: 10.1186/s12889-024-20640-4 (PMC11575446; doi:10.1186/s12889-024-20640-4)
Supplement: Supplementary file 1 — Supplementary Material 1 [file 12889_2024_20640_MOESM1_ESM.pdf]

IRON DEFICIENCY ANEMIA AND ITS ASSOCIATION WITH COGNITIVE FUNCTION AMONG ADOLESCENTS IN THE ASHANTI REGION - GHANA

Kindly take a few moments to respond to the following questions about "**Iron Deficiency Anemia and its Association with Cognitive Function among Adolescents in the Ashanti Region - Ghana".** The study is solely for academic purposes, and any information you offer on this form will be kept in strict confidence. For more information about this survey, please, contact the form's administrator.

CASE ID ……………………. **DATE…………………**

SECTION 'A': SOCIODEMOGRAPHIC & LIFESTYLE DATA

1. Age …………………………………….. (years)
2. Weight…………………………………………. (kg)
3. Height………………………………………….. (m)
4. BMI …………………………………………….. (Kg/m^2)
5. Gender
   A. Male [ ] B. Female [ ]
6. Nationality
   A. Ghanaian [ ] B. Non- Ghanaian [ ]
7. Residency
8. A. Rural [ ] B. Urban [ ]
9. Religion
   A. Christian B. Islamic [ ] C. Traditionalist [ ]
   D. Other (Please specify) ……………………………………………
10. Father's Highest Educational status
    A. Primary [ ] B. Junior High [ ] C. Secondary [ ]

    D. Tertiary [ ] E. Others (Please specify)
    …………………………………………………………

11. Mother’s Highest Educational status

B. Primary [ ] B. Junior High [ ] C. Secondary [ ]

E. Tertiary [ ] E. Others (Please specify)

………………………………………………………

12. Father’s Occupation

A. Businessman/ Trader [ ] B. Civil Servant [ ] C. Daily Labourer [ ]

D. No Work [ ] E. Other (Please specify) …………………………………

13. Mother’s Occupation

B. Business Woman/ Trader [ ] B. Civil Servant [ ] C. Daily Labourer [ ]

D. No Work [ ] E. Other (Please specify) …………………………………

14. Family Size

A. Small (1 -2 children) [ ] B. Medium (3 – 5 children) [ ]
C. Large > 6 children [ ]

SECTION ‘B’ CLINICAL DATA

15. Any history of Parasitic Infections

A. Yes [ ] B. No [ ]

16. If yes, when was the last time you got infected and treated.

A. A week ago [ ] B. 2 weeks [ ] C. Last month [ ]

D. Last three (3) months

17. Have you taken any type of de-wormer medication before?

A. Yes [ ] B. No [ ]

18. If yes when was the last time

A. A week ago [ ] B. Two weeks C. Last month [ ]

D. Three months ago [ ] E. Six months ago [ ] F. More than a year [ ]

19. Family History of any diseases

A. Sickle cell [ ] B. Haemolytic disease [ ] C. Others (please specify)
……………

20. Are you on Hematinic or any other Medication?

A. Yes [ ] B. No [ ] C. Others (please specify)……………………………….

21. If yes when was the last time you took these medications

A. Today [ ] B. Yesterday [ ] c. Last week [ ]

D. Last month E. Last three (3) months [ ]

SECTION ‘C’ DIETARY HABITS

Pls select the appropriate answer that is in consistence with your daily meal

22. Calories / Carbohydrate

A. Non Consumer [ ] B. Low Consumer [ ] C. Consumer [ ]

23. Fresh Vegetables

A. Non Consumer [ ] B. Low Consumer [ ] C. Consumer [ ]

24. Fruits

A. Non Consumer [ ] B. Low Consumer [ ] C. Consumer [ ]

25. Dairy Products

A. Non Consumer [ ] B. Low Consumer [ ] C. Consumer [ ]

26. Protein Source Food

A. Non Consumer [ ] B. Low Consumer [ ] C. Consumer [ ]

27. Fats / Oils

A. Non Consumer [ ] B. Low Consumer [ ] C. Consumer [ ]

SECTION ‘D’ SLEEPING HABITS

28. Are you able to sleep well

A. Yes [ ] B. No [ ]

29. Pls specify the number of hours you sleep daily

………………………………………………………………………………………..

SECTION 'E' LEARNING HABITS

30. Do you have a smooth study in all your subjects

A. Yes [ ] B. No [ ]

31. Do you have learning difficulties in any of your subjects

A. Yes [ ] B. No [ ]

32. How many mins/hours do you use for your personal studies

…………………………………………………………………………………………
………………

33. Are you able to remember things learnt very fast

A. Yes [ ] B. No [ ]

34. How will you rate/score your learning capacity/ memory

A. Excellent [ ] B. Very Good [ ] C. Good [ ]
C. Moderate [ ] E. Weak [ ] F. Not too Bad [ ]

SECTION 'F' MENSTRUAL PROFILE

Strictly for only ladies

35. Currently are you in your menses

A. Yes [ ] B. No [ ]

36. Describe your menstrual pattern

A. Regular [ ] B. Non Regular [ ]

37. How many days do the menstruation last

A. 2 days [ ] B. 3 days [ ] C. 4 days [ ] D. 5 days [ ] E. 6 days [ ] F. 7 days [ ] G. More than a week [ ]

38. Pls describe the quantity of blood flow

A. Very Heavy [ ] B. Heavy [ ] C. Moderate [ ] D. Light [ ]

SECTION ‘G’ COGNITIVE PERFORMANCE TEST-TONI 4

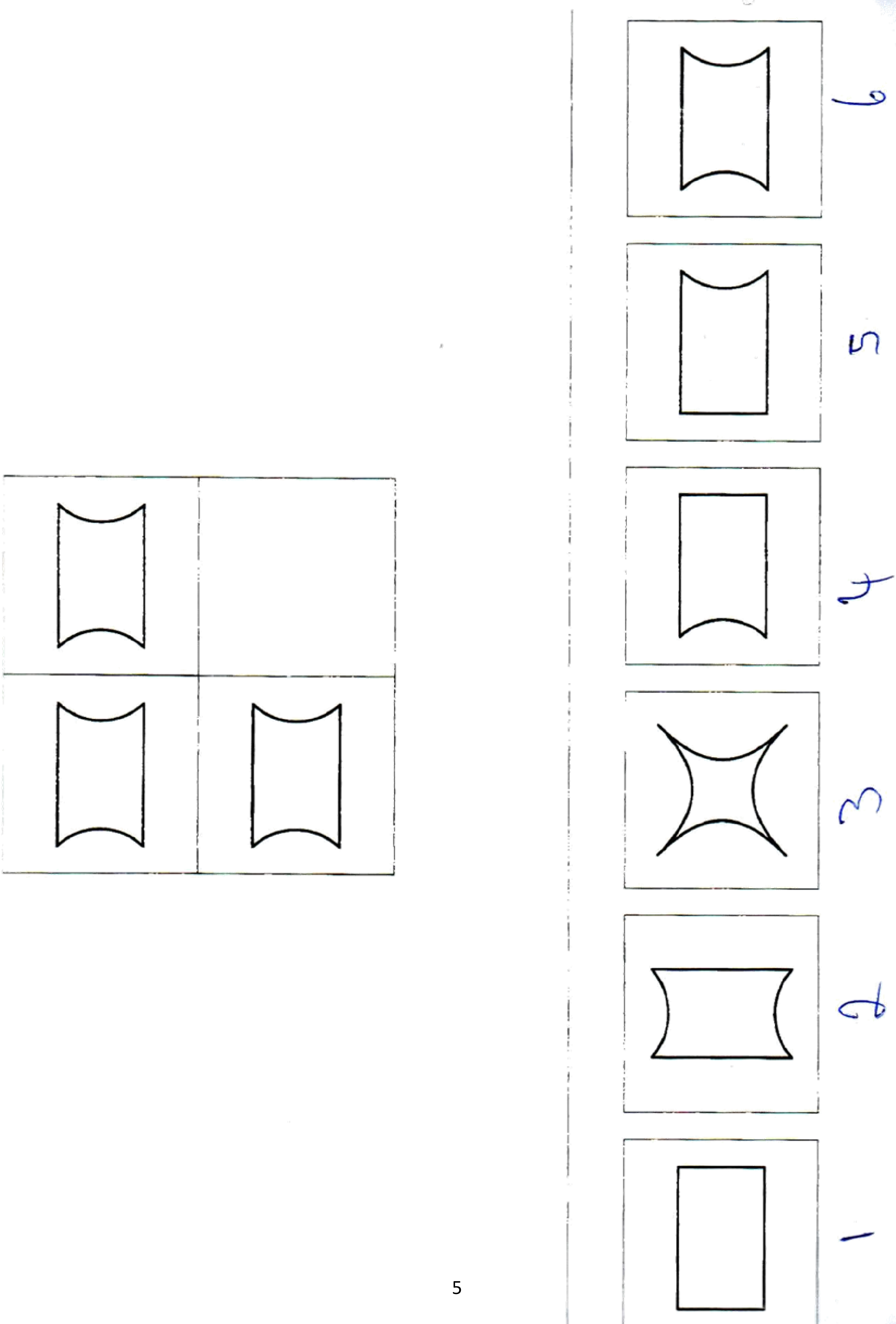

A5

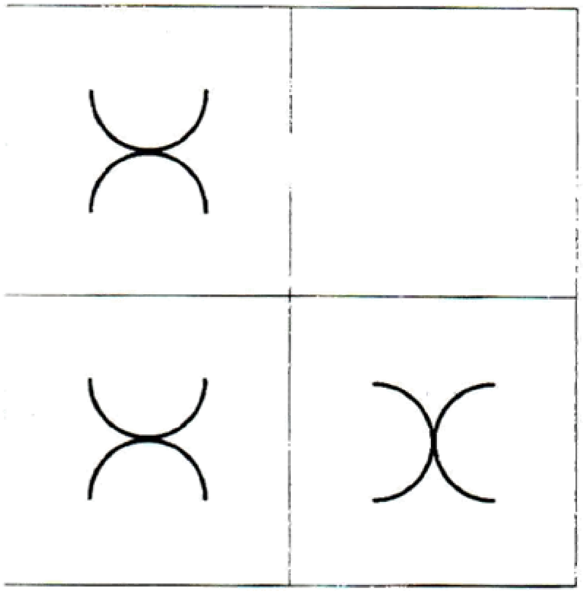

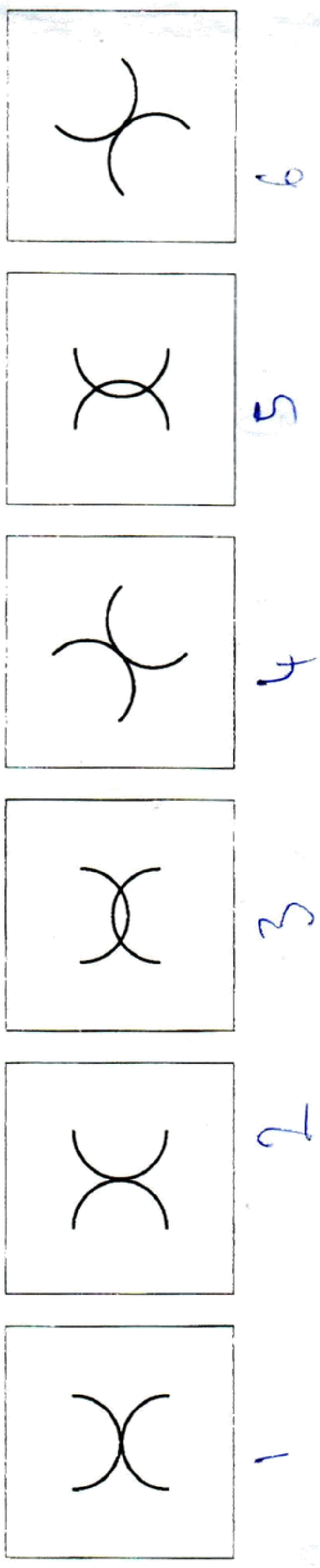

A6

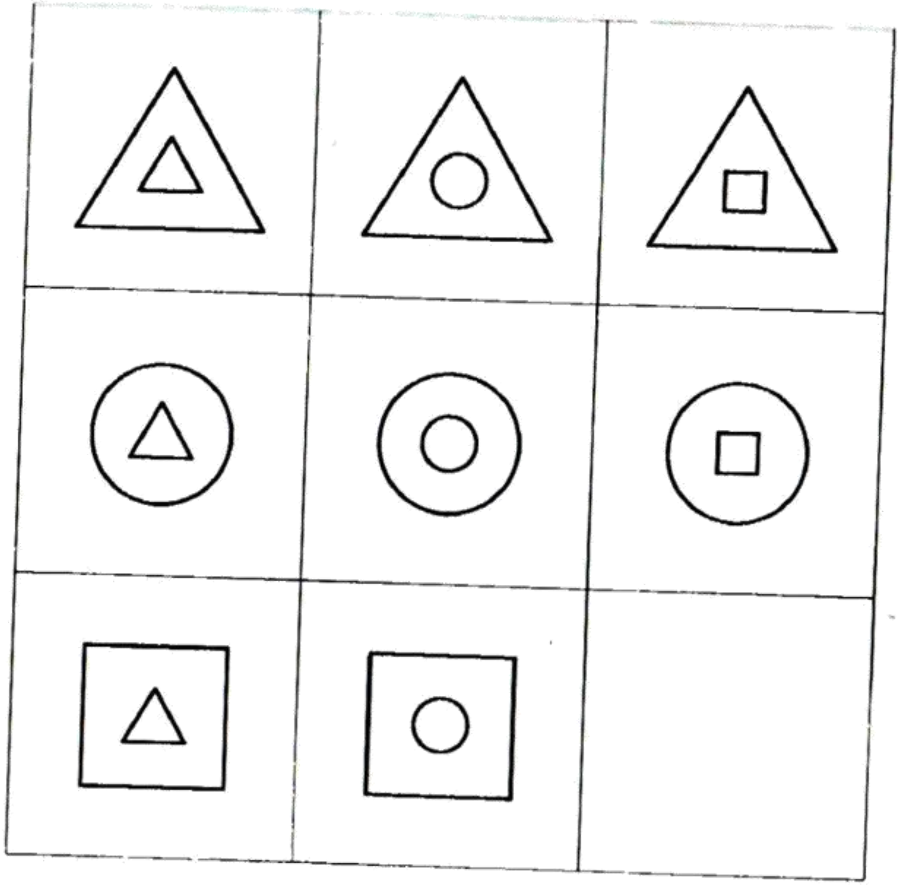

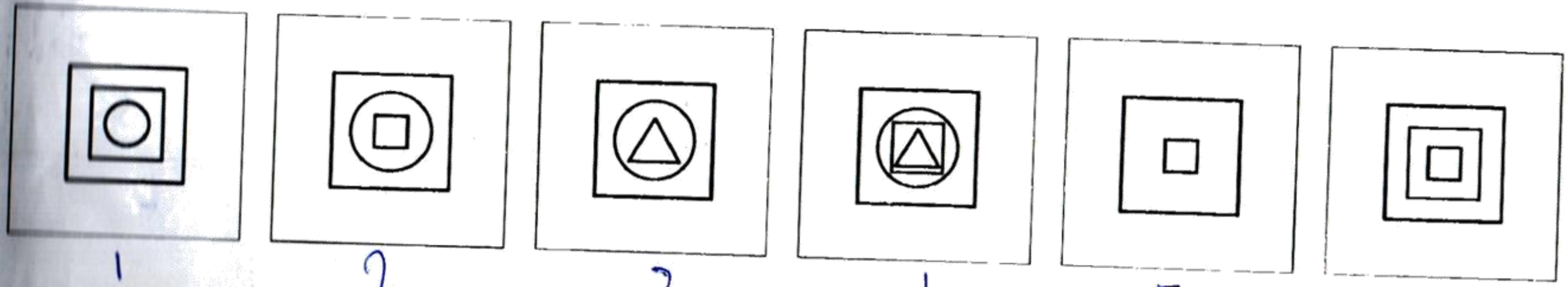

1 2 3 4 5 6

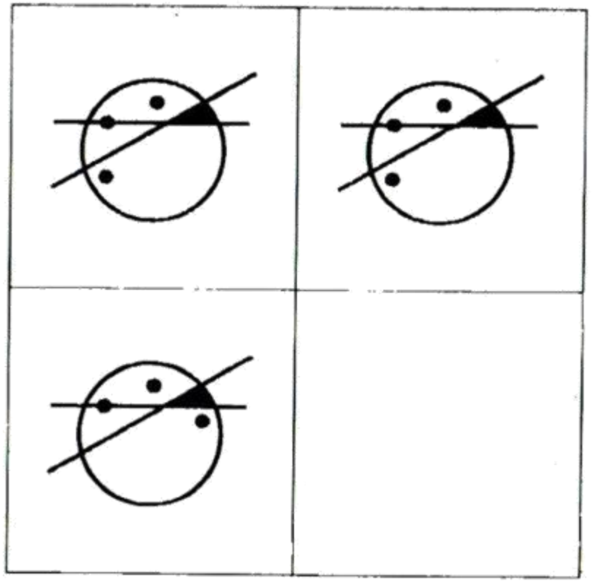

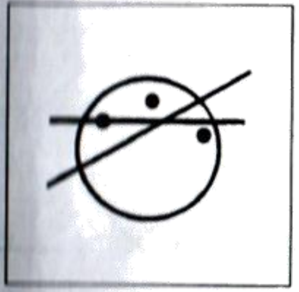

1

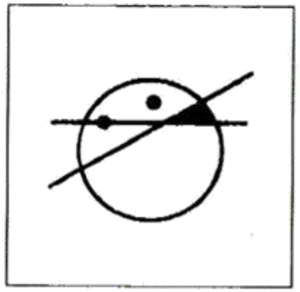

2

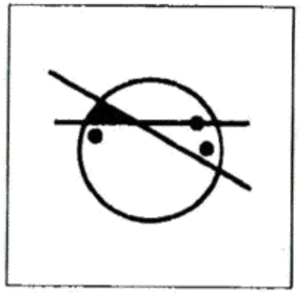

3

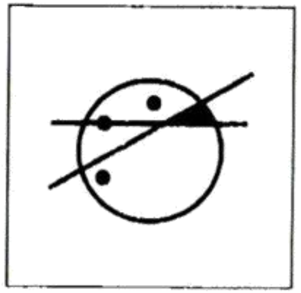

4

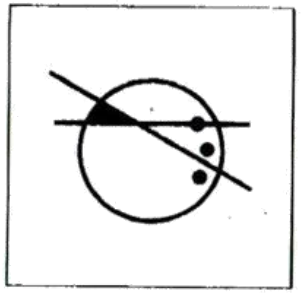

5

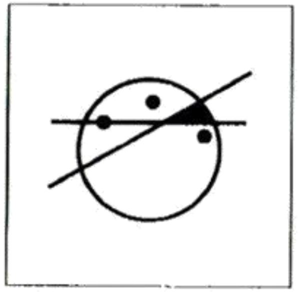

6

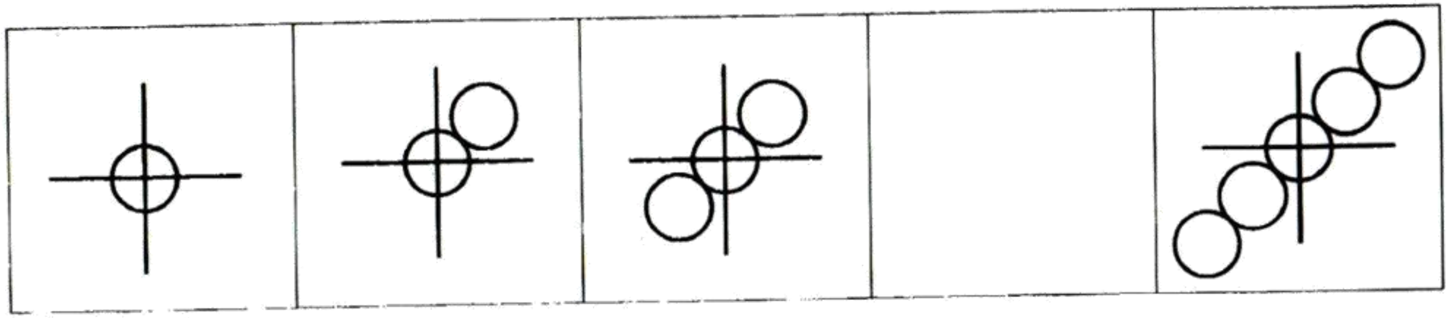

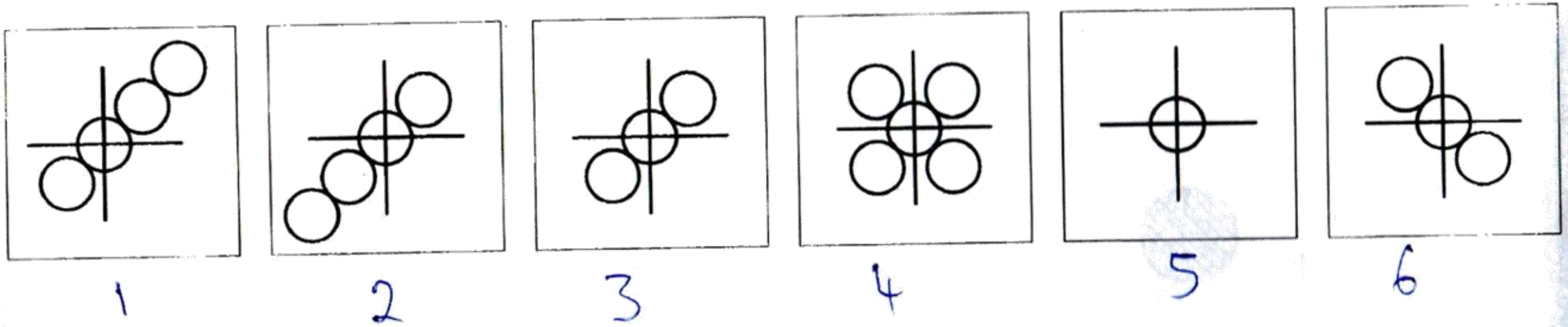

A22

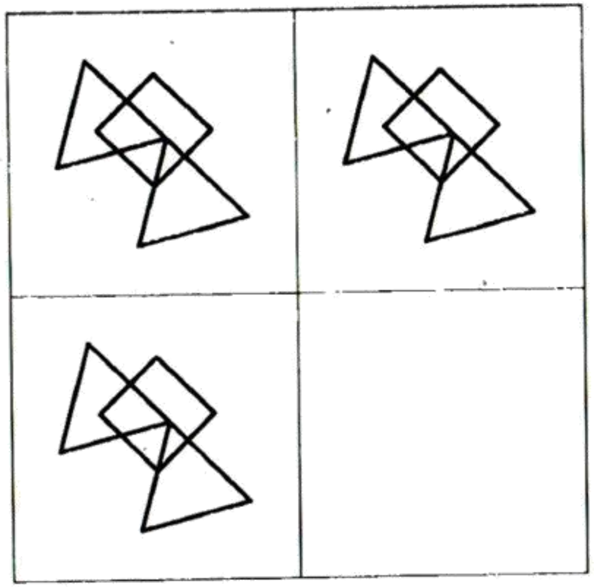

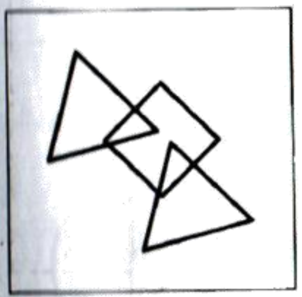

1

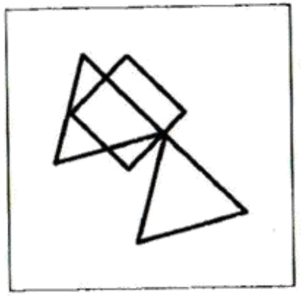

2

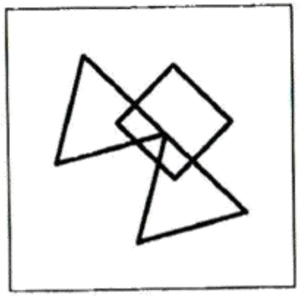

3

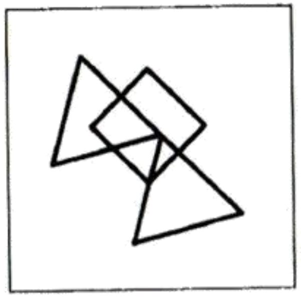

4

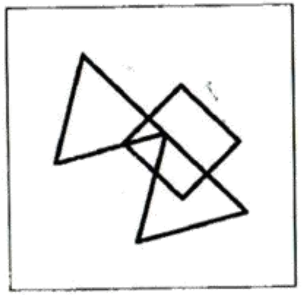

5

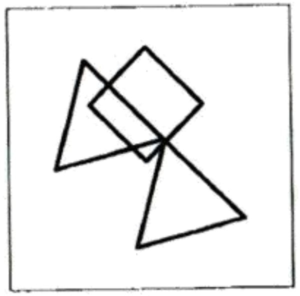

6

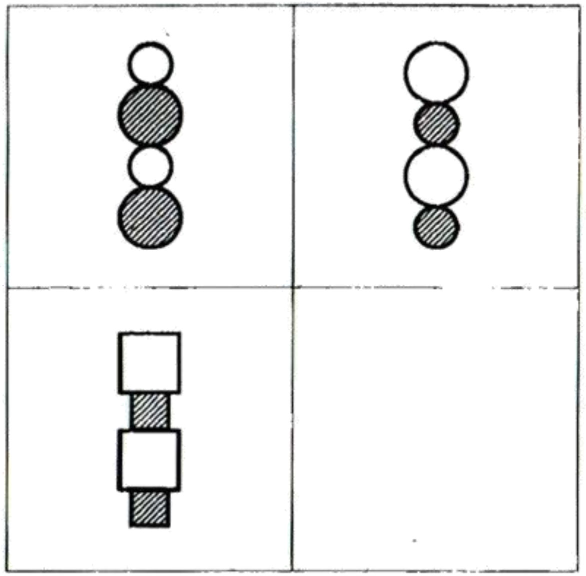

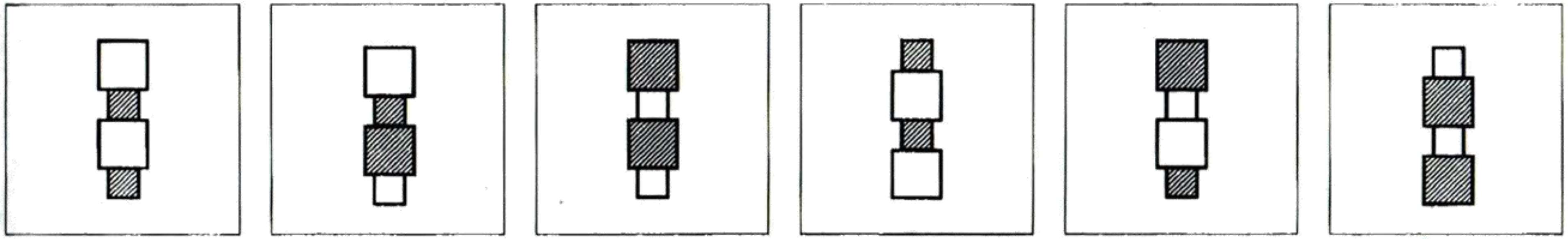

1 2 3 4 5 6

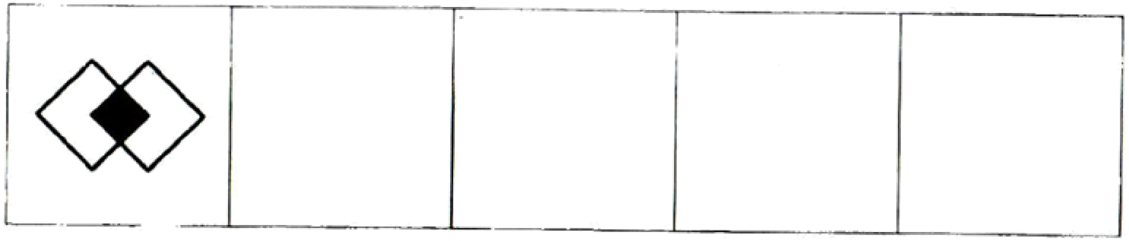

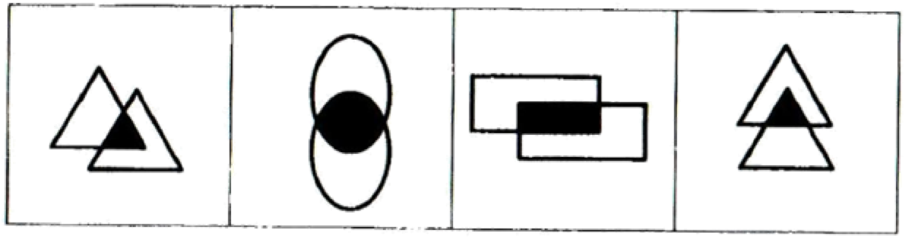

1

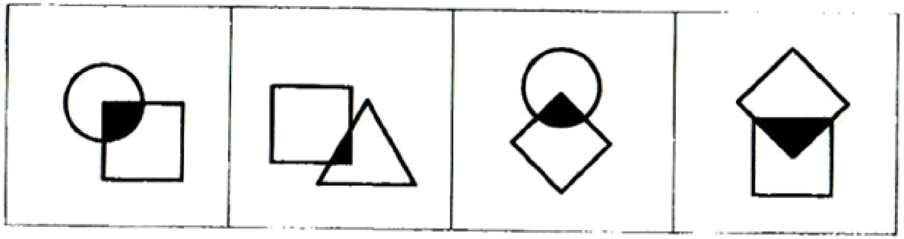

2

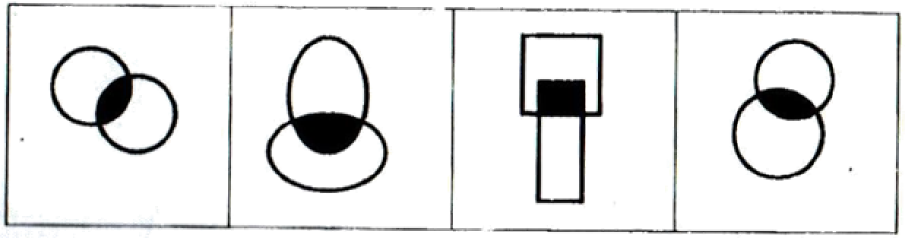

3

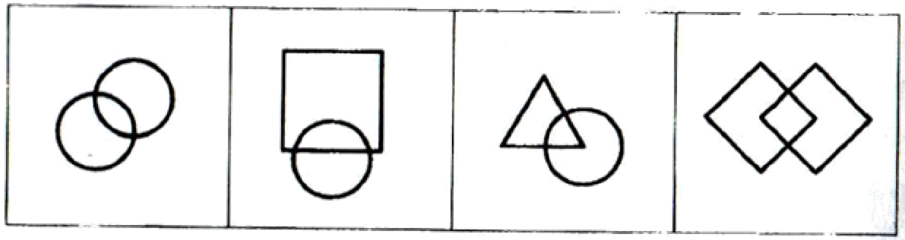

4

A36

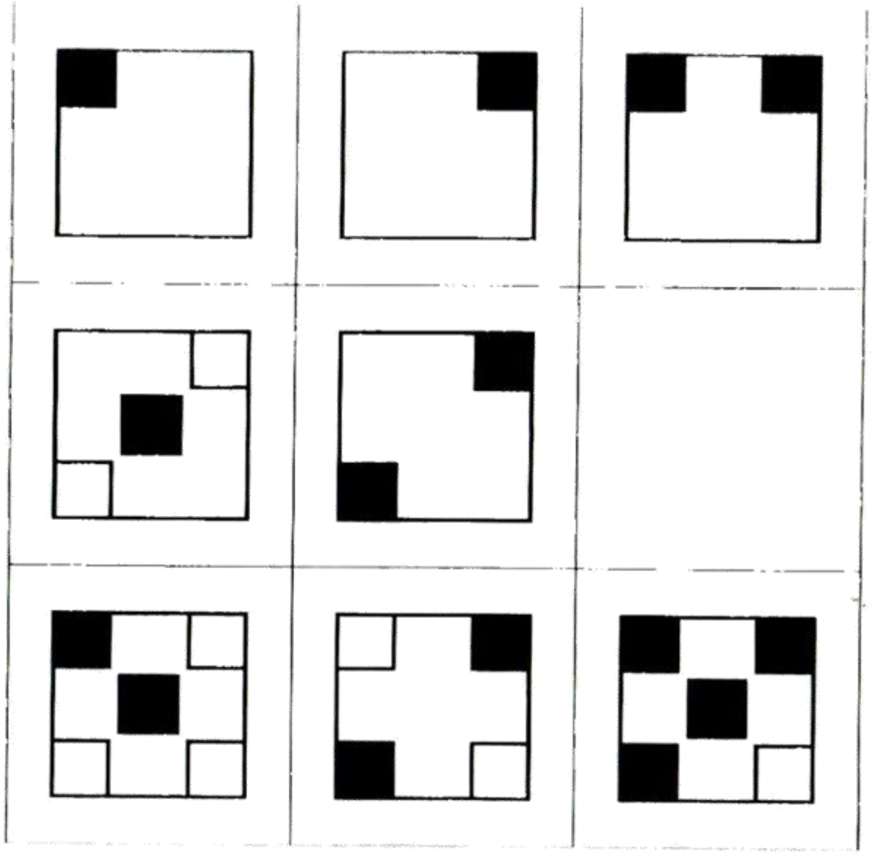

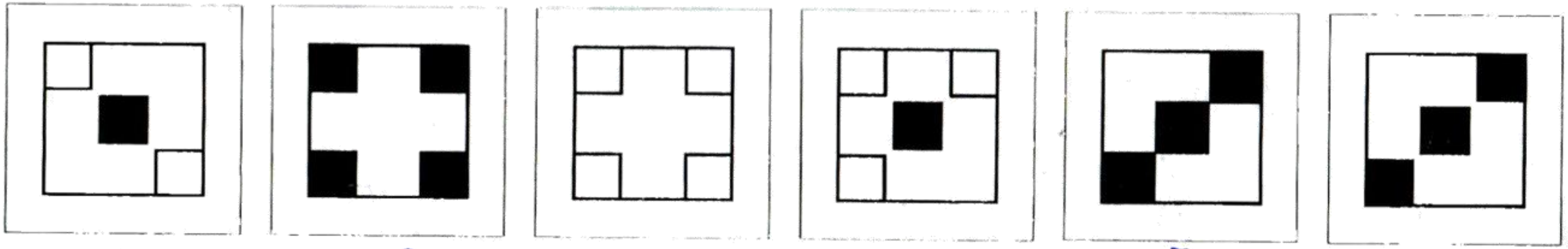

1 2 3 4 5 6

A41

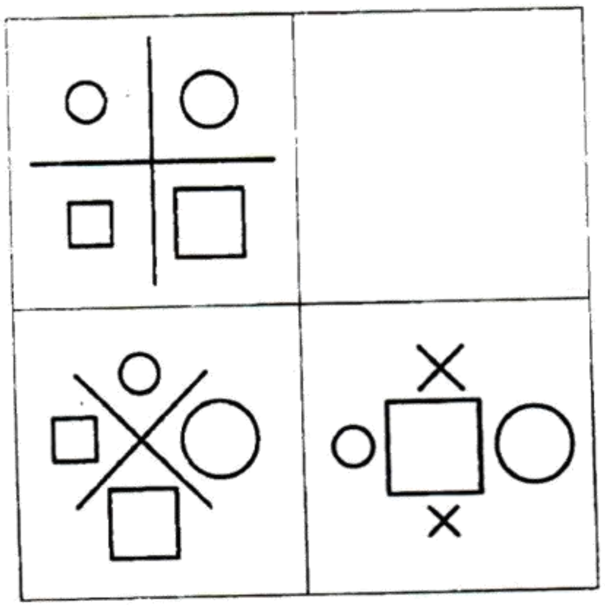

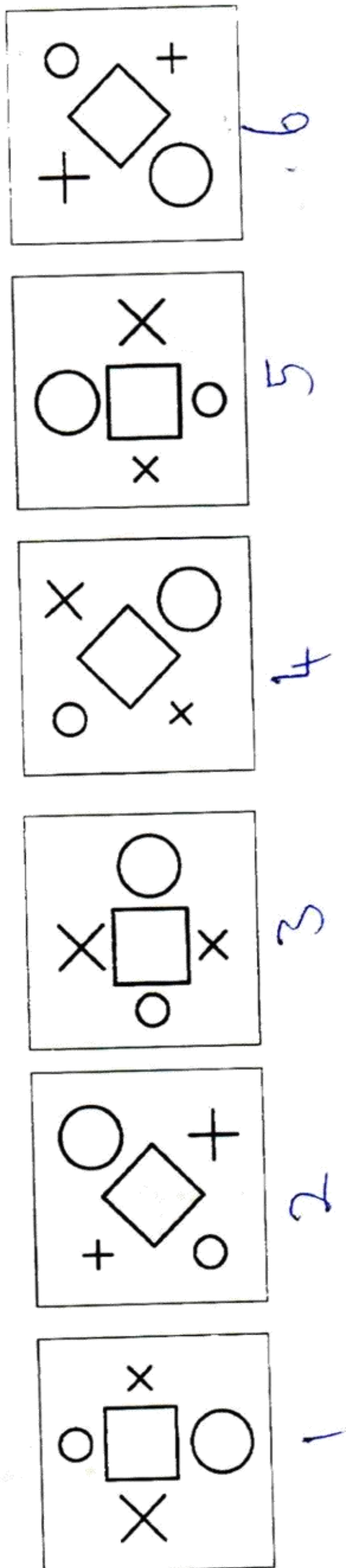

A45
